# Data resource profile: JMDC claims database sourced from health insurance societies

**DOI:** 10.1002/jgf2.422

**Published:** 2021-02-14

**Authors:** Katsuhiko Nagai, Takashi Tanaka, Norihisa Kodaira, Shinya Kimura, Yoshimitsu Takahashi, Takeo Nakayama

**Affiliations:** ^1^ JMDC Inc. Tokyo Japan; ^2^ Department of Health Informatics Kyoto University School of Public Health Kyoto Japan

**Keywords:** database, health insurance, health insurance societies, Japan

## Abstract

JMDC, Inc. (JMDC) has created a database, using data collected from health insurance societies in Japan, consisting of ledgers of insureds, claims (for hospitalization, outpatient treatment, drug preparation, and dental treatment), and health checkup results. The earliest data are from the claims in January 2005, except dental claims from December 2009 and health checkup results from April 2008. Currently (the end of June 2020), the number of insureds included is approximately 9.8 million. This database is unique for Japan and has the following characteristics: (a) the basic population can be ascertained; (b) standardization is carried out using a dictionary; and (c) anonymized individual IDs can be followed on the basis of a time‐series over various periods, with the earliest starting date being January 2005. However, it has certain limitations, in that the disease status and test results cannot be ascertained, and there is insufficient access to data for elderly people.

## Graphical abstract

JMDC, Inc. (JMDC) has created a database, using data collected from health insurance societies in Japan, consisting of ledgers of insureds, claims (for hospitalization, outpatient treatment, drug preparation, and dental treatment), and health checkup results. This database is unique for Japan and has the following characteristics: (a) the basic population can be ascertained; (b) standardization is carried out using a dictionary; and (c) anonymized individual IDs can be followed on the basis of a time‐series over various periods, with the earliest starting date being January 2005

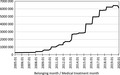

## 1 DATA RESOURCE BASICS

Japan has health insurance provided by the social insurance system, and this health insurance is provided by multiple bodies, on the basis of occupation, geography, and age (eg, elderly, geriatric), including health insurance associations which cover medical costs except for employment‐related injury of people less than 75 years old, primarily those who are employees of large businesses, and their dependents.^1^

**FIGURE 1:**
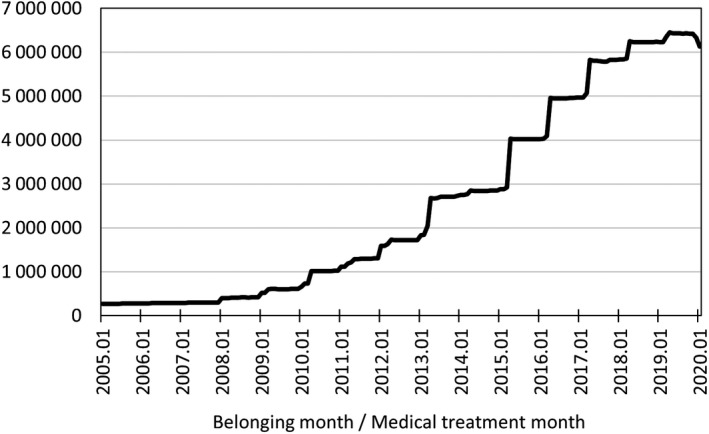
Numbers of insured persons whose data can be accessed each month

JMDC, Inc. (JMDC) has created a database, detailed below, using data collected from health insurance societies, as well as the databases sourced from medical institutions.^1^ We describe the detailed information below although it is partly redundant with the information of the medical institutions' databases^1^:

1. The collected data consist of ledgers of insured persons, claims (for hospitalization, out‐patient treatment, drug preparation, and dental treatment), and health checkup results. Ledgers of insured persons include information about all persons insured by health insurance societies. Claims include information about medical expenses for which health insurance societies have been invoiced by medical institutions and about the insured persons who incurred the medical expenses. Health checkups are carried out so that health insurance societies can ascertain the health statuses of insured persons, and special health checkups are required to be conducted with the aim of preventing lifestyle‐related diseases in insured persons aged 40‐74.
2. JMDC provides services of data analysis of actual conditions such as medical cost analysis by comparison with other insurers and extraction of high‐risk persons to health insurance societies distributed throughout Japan. Some of these health insurance societies agree to provide anonymously processed data of employees working at all branches and offices in Japan of the companies covered by the societies and their families, which do not include area information to third parties. Data are collected from these health insurance societies. The specific names and geographical information of included societies are not disclosed from JMDC because of their policy.
3. Data are collected monthly, and collected data are added to the database approximately five months after the treatment date. Data are currently still being added to the database.
4. In order to protect personal information, the collected data are added to the database as information that has been anonymized in accordance with Clause 2:9 of the Law for Protection of Personal Information, on the basis of personal IDs with which individuals cannot be identified. The personal ID is set by health insurance societies as an individual unit for managing insured person ledgers and claims, making it possible to recognize the same individual, if insured by the same health insurance society, even if treated by different medical institutions.
5. The earliest data in the database that can be accessed are from the claims in January 2005. However, dental claims are included only from December 2009, and health checkup results only from April 2008.
6. Currently (the end of June 2020), the number of insured persons included in the database, including those withdrawn at some stage, is approximately 9.8 million. As persons aged 75 or more cannot have health insurance administered by health insurance societies, all persons included in the database are aged 0 to 74.
7. The monthly numbers of insured persons whose data can be accessed are shown in Figure 1. The number of insured persons tends to increase with increasing number of health insurance societies collecting data. Currently (the end of June 2020), the number of health insurance societies collecting data is 216, and the number of insured persons is approximately 6.1 million.
8. Currently (the end of June 2020), the number of hospital department and drug preparation claims included in the database is approximately 370 million, and the number of actual patients, that is, the number of insured persons for whom at least one hospital department and/or drug preparation claim is released, is approximately 8.9 million.
9. In the last 5 calendar years, the annual withdrawal rates by month from this database were about 8%–10%. It should be noted that those who moved to other included societies were also counted as withdrawals in this calculation, because it is difficult to determine the same person who has moved to another society.

Research carried out using this database includes the following:

1. Drug utilization surveys (longitudinal and transverse)^2^, ^3^, ^4^, ^5^, ^6^, ^7^
2. Drug risk assessment^8^, ^9^, ^10^
3. Evidence‐practice gap^11^, ^12^, ^13^, ^14^, ^15^
4. Effectiveness of intervention^16^, ^17^, ^18^
5. Cost and economic analysis^19^, ^20^, ^21^, ^22^
6. Health checkup^23^, ^24^, ^25^, ^26^
7. Validation^27^, ^28^

## 2 DATA COLLECTED

All data collected from health insurance societies are anonymized on the basis of personal ID so that individuals cannot be identified.

Nonstandardized data are standardized using a dictionary like the databases sourced from medical institutions JMDC created.^1^

Abnormal values, time‐series consistency, etc., are verified independently by the JMDC. Missing values are left as they are but are relatively small in claims because the claim data submitted to the claims processing and payment organization are error checked in the external examination processes. However, it should be noted that the health checkup results have many missing values because JMDC does not collect health checkup results from all the health insurance societies JMDC collects data.

The database consist of (a) tables for the population; medical institution; claims; claims: injury/disease; claims: drugs; claims: medical care activities; claims: treatment materials; and health checkup results; and (b) master data for injuries/diseases, drugs, medical care activities, etc., including standardized medical terms. Explanations of all the tables, and examples of fields are shown in Table 1.

**TABLE 1:** Database of data from health insurance societies

Table	Explanation	Field example
Population	Information about insured persons	Month and year of birth, Gender code, Code for whether he/she is employee or employee's family, First month observable, Last month observable, Flag for withdrawal while in progress, Flag for termination due to death
Medical institutions	Information about medical institutions	Range in number of beds at medical institutions, Management body code, Flag for designated cancer care hospitals
Claims	Claim information summary (data provided to claims processing and payment organization)	Month and year of medical care, Code for type of claim (hospitalized, outpatients, DPC, etc), Actual number of days of medical care, Hospital admission date, Discharge date, Number of points (medical expenses), Diagnosis procedure combination (DPC) code
Claims: injury/disease	Information about injury/disease of hospitalized patients and outpatients (data provided to claims processing and payment organization)	First date of medical care of injury/disease, The 10th revised version of the International Statistical Classification of Diseases and Related Health Problems (ICD‐10) classification code, Injury/disease code, Flag for principal injury/disease, Flag for injury/disease which used the most resources, Flag for suspicious injury/disease, Outcome category code
Claims: drugs	Drug information relating to hospitalized patients and outpatients (data provided to claims processing and payment organization)	Prescription date, Drug preparation date, The European Pharmaceutical Market Research Association (EphMRA) anatomical therapeutic chemical (ATC) classification code, World Health Organization (WHO) ATC classification code, Japanese Ministry of Health, Labor, and Welfare (MHLW) drug code, Drug code for electronic claim processing, Daily dose per prescription, Dose unit, Number of days of administration per prescription, Dosage, Number of points (medical expenses), Flag for generics, Flag for drugs to take as necessary, Dosage form classification code
Claims: medical care activities	Information about medical care activities of hospitalized patients and outpatients (data provided to claims processing and payment organization)	Medical care activities date, Medical care activities code, Number of times performed, Number of points (medical expenses)
Claims: treatment materials	Information about materials for hospitalized patients and outpatients (data provided to claims processing and payment organization)	Treatment date, Material code, Number of times performed, Number of points (medical expenses)
Health checkup results	Information about health checkup results	Health checkup date, Body mass index (BMI), Abdominal circumference (AC), presence of medical history, Presence of subjective symptoms, Presence of objective symptoms, Systolic blood pressure (SBP), Diastolic blood pressure (DBP), Category of time of blood collection, Triglyceride (TG), HDL cholesterol, LDL cholesterol, GOT (AST), GPT (ALT), γ‐GT (γ‐GTP), Fasting blood sugar (FBS), Casual blood sugar (CBS), Hemoglobin A1c (HbA1c), Code for urinary sugar, Code for uric protein, Serum uric acid, Serum creatinine, Total cholesterol, Hematocrit, Hemoglobin content, Erythrocyte count, Electrocardiographic findings (signs present or absent), Optic fundus examination (Keith‐Wagener classification), Optic fundus examination (Scheie's classification: H), Optic fundus examination (Scheie's classification: S), Optic fundus examination (SCOTT classification), Smoking habit, Eating mode 1 (fast eating, etc), Eating mode 2 (before sleeping), Eating mode 3 (between meals), Eating habit, Alcohol consumption presence or absence, Alcohol consumption quantity, Medication 1 (blood pressure), Medication 2 (blood sugar), Medication 3 (cholesterol), Medical history 1 (cerebrovascular disease), Medical history 2 (cardiovascular), Medical history 3 (renal failure / artificial dialysis), Anemia, Weight change since age 20, Exercise habit, Walking or other physical activity, Walking speed, Weight change in a year, Masticatory function, Improvement in living habits, Health guidance wanted or not wanted

## 3 DATA RESOURCE USE

The following analyses can be carried out using this database:

1. The data are from a complete‐enumeration survey of the population, including healthy people. Therefore, for certain diseases, chosen on a discretionary basis, the prevalence, incidence rate, specific treatment rate, medical expenses per patient, and number of days with hospital visits or admissions can be calculated.
2. The data were from a survey that followed the patient longitudinally. Therefore, it is possible to analyze sequential data (hospital visit and admission histories, and numbers of health checkups).
3. It is possible to conduct epidemiological studies with the health checkup results as exposure, and information obtained from the claims as the endpoints. In this case, it will be preferable to validate some sort for the disease name in the claims.
4. Deaths recorded in the ledger of insured persons can be used as an endpoint for epidemiological studies. In the case of deaths in the outcome information in claims, validation studies have shown the sensitivity, specificity, and positive predictive value in relation to hospitalized deaths in patients of aged over 65‐74 be approximately 95%.^28^

The most recent reference lists for this database of data from health insurance societies are summarized on the publication lists page on the JMDC website (https://info.jmdc.co.jp/jrda/). Please note that these lists correspond to two databases of JMDC, this database of data from health insurance societies and the other database of data from medical institutions,^1^ and currently most are from this database of data from health insurance societies.

## 4 STRENGTHS AND WEAKNESSES

From a clinical point of view, this database covers all claims for all members of the health insurance societies JMDC collects data and enables patient‐based tracking of visits and treatment flows even if the patient was transferred to another hospital during treatment or was completely cured, unlike the databases sourced from medical institutions. In addition, it is possible to grasp health conditions of patients to some extent, using health checkup results in this database. Examples of research using this database are described in the “Data resource use” section. Currently (the end of June 2020), there are about 250 published papers out of studies using this database, as far as we know.

This database offers the following advantages:

1. As the master data are accorded after standardization of all data, researchers need almost no data pretreatment for analysis.
2. Data are available about the populations insured by health insurance societies; therefore, it is possible to ascertain the prevalence and incidence rates for different genders and age‐groups.
3. In the case of people insured by the same health insurance society (but not in other cases), all claims are handled in a centralized manner. Therefore, when the same individual is treated by more than one medical institution, the information about him/her can be determined comprehensively.
4. In addition to information about claims, health checkup data are available.

However, it has the following disadvantages:

1. As the data source is health insurance societies, there are few data from people aged over 65 and none from people aged over 75.
2. If a member of health insurance society withdraws, the data are discontinued.
3. As the claim data are for the purpose of medical expense invoicing, the disease names used are those used for insurance purposes; so, if the disease has to be defined, a contrivance of some sort is required.
4. No clinical laboratory test values are included, so the ability to determine the disease severity and clinical presentation is limited.
5. No data are available for treatment outcome, such as the status at discharge.

**FIGURE 2:**
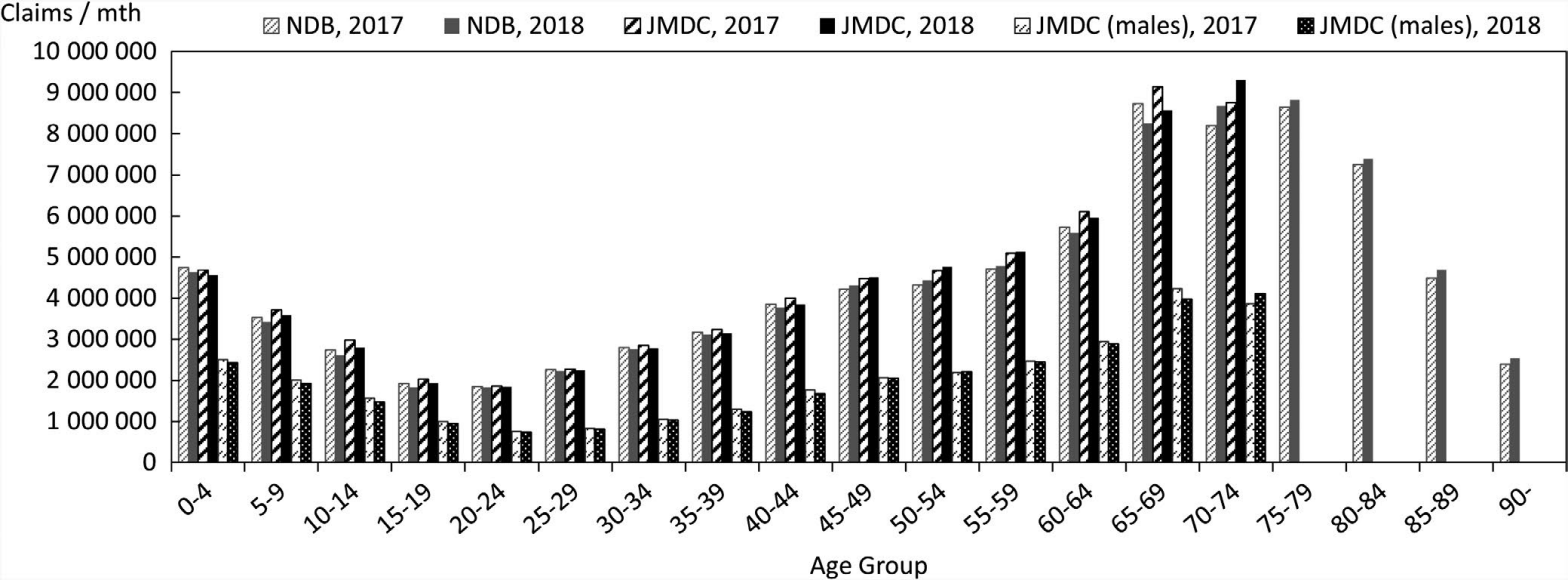
Comparison between scaled‐up data from health insurance societies and publicly available data (total numbers of hospitalized and outpatients for each gender and age‐group)

**FIGURE 3:**
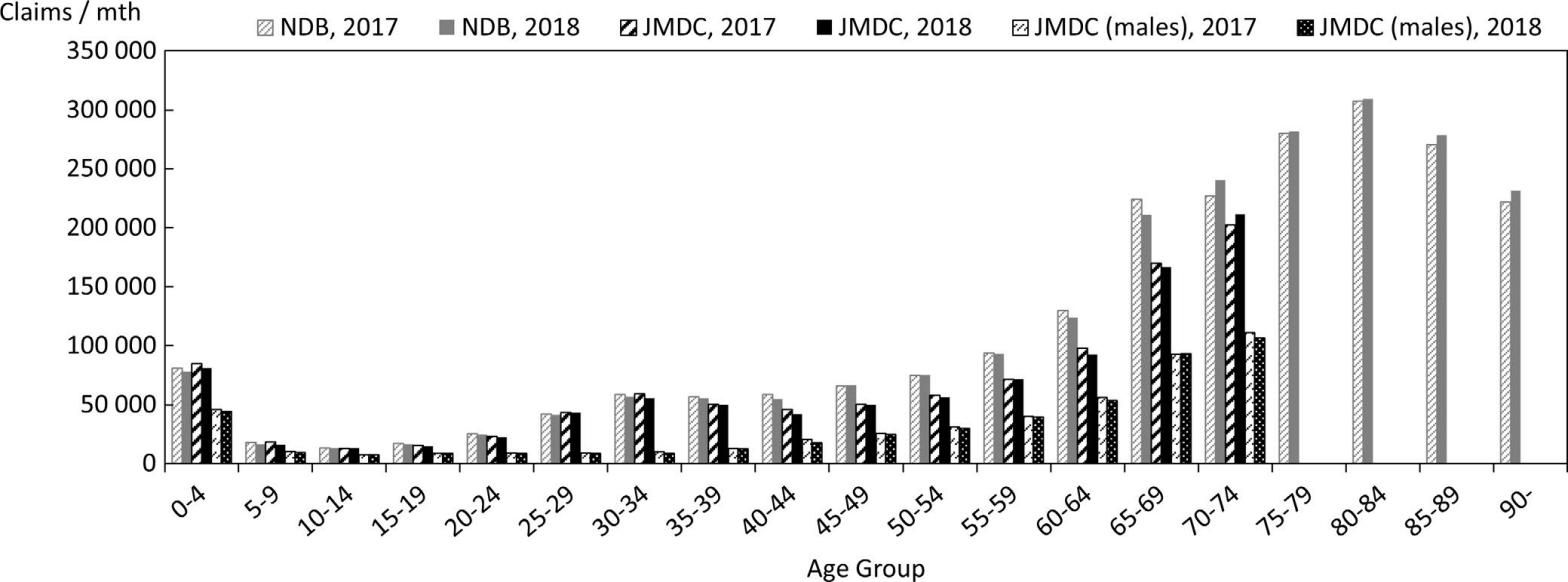
Comparison between scaled‐up data from health insurance societies and publicly available data (numbers of hospitalized for each gender and age‐group)

**FIGURE 4:**
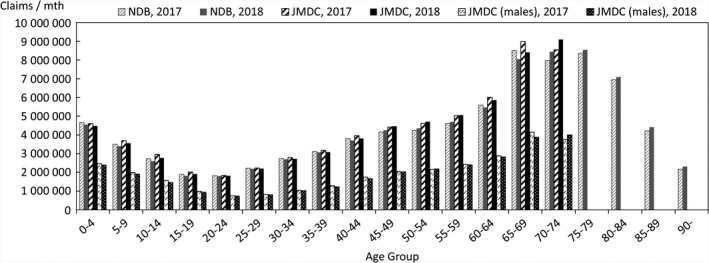
Comparison between scaled‐up data from health insurance societies and publicly available data (numbers of outpatients for each gender and age‐group)

**FIGURE 5:**
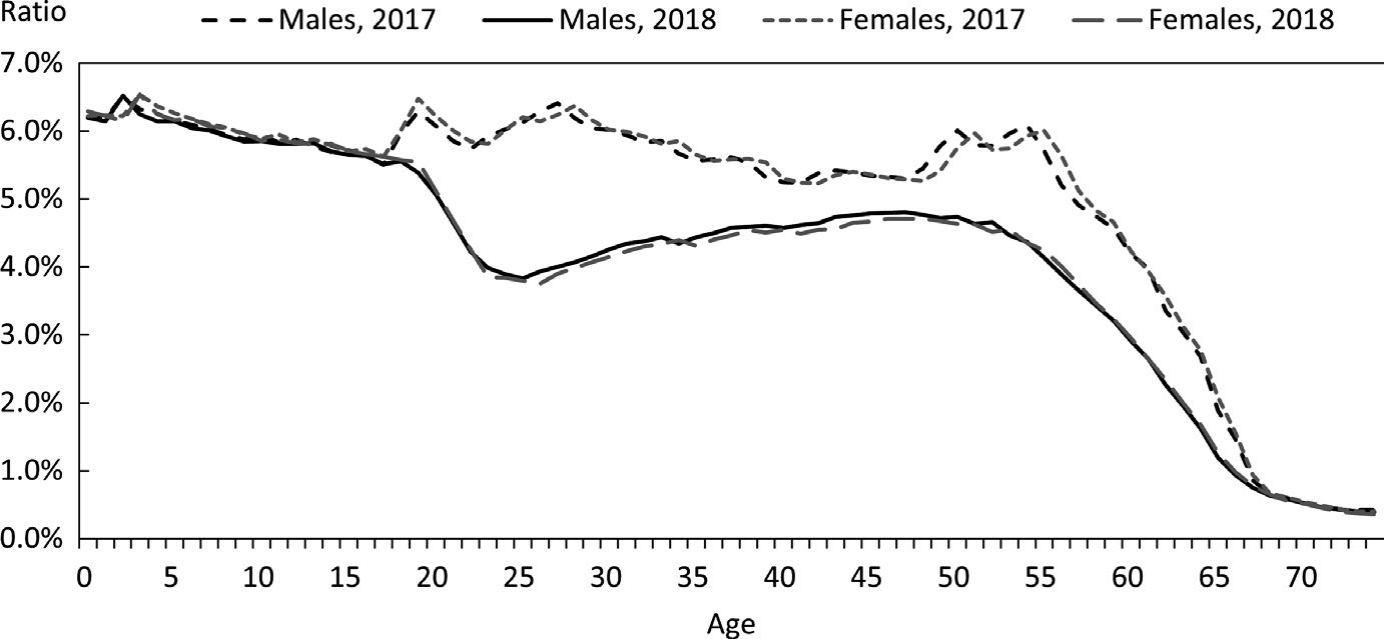
Ratios used for comparison of data from health insurance societies relative to publicly available data (database population relative to total population of Japan)

In order to quantify the above characteristics, comparisons were made with publicly available data. The data used for the comparison were the numbers of claims in the June 2017 and June 2018 reviews in the MHLW's Statistics of Medical Care Activities in Public Health Insurance, which were aggregates of the National Database of Health Insurance Claims and Specific Health Checkups (NDB). The JMDC numbers of claims to be compared with them were obtained by scaling up the numbers of claims for treatment during May 2017 and May 2018 in this database of the data from health insurance societies by the relative numbers of persons of each gender and age‐group in the total population of Japan and in the insured person population of this database at the beginning of October 2017 and October 2018. In this context, the publicly available data for the total population of Japan were taken to be the total populations of each gender and age‐group, as of October 1 each year, shown in Table 1 (Population classified by age [each year] and gender, and population gender ratios, in total population and population of Japanese people) in the Population Estimates of the Ministry of Internal Affairs and Communications. The results for each gender and age‐group are shown in Figures 2, 3, 4, in which the disposition of males in the JMDC numbers is also shown. The ratios used for scaling up are shown in Figure 5.

**FIGURE 6:**
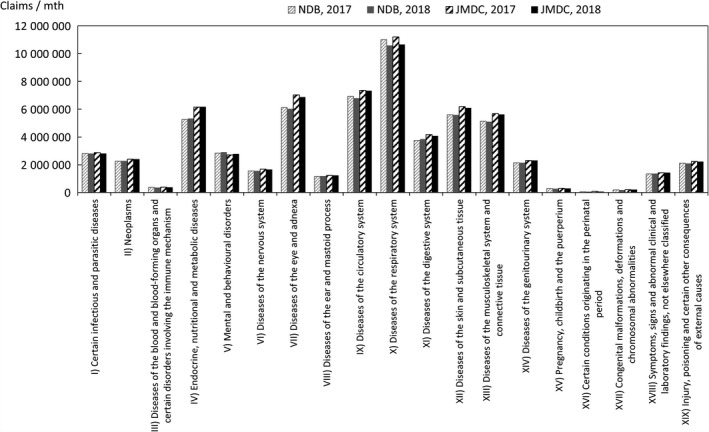
Comparison between scaled‐up data from health insurance societies and publicly available data (total numbers of hospitalized and outpatients for each chapter of ICD‐10)

**FIGURE 7:**
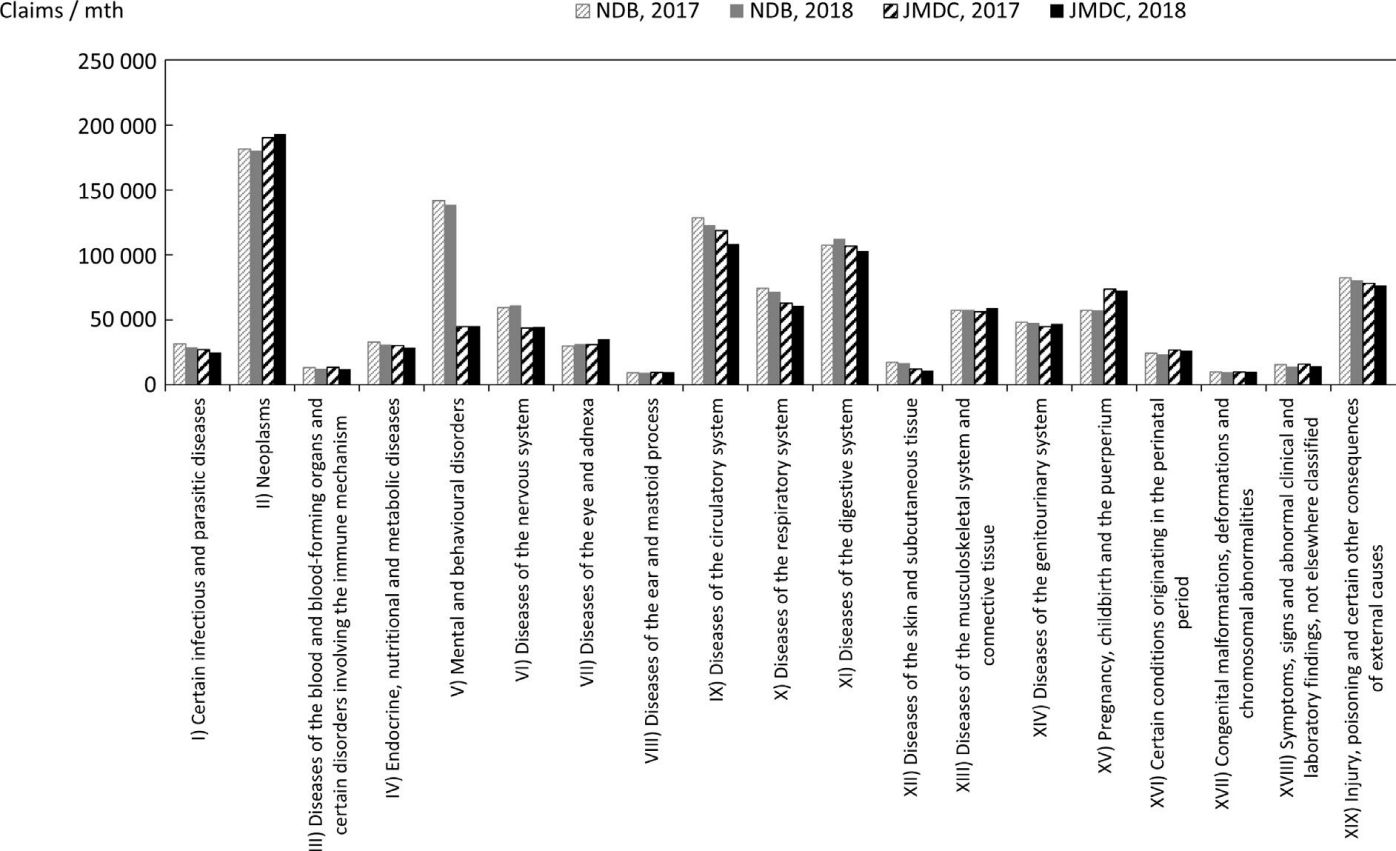
Comparison between scaled‐up data from health insurance societies and publicly available data (numbers of hospitalized for each chapter of ICD‐10)

**FIGURE 8:**
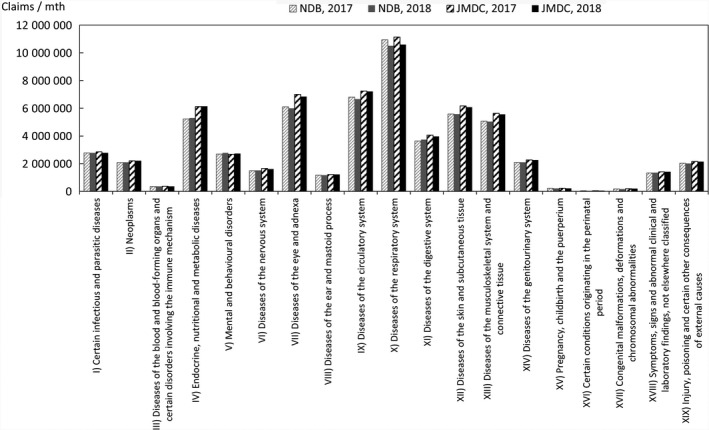
Comparison between scaled‐up data from health insurance societies and publicly available data (numbers of outpatients for each chapter of ICD‐10)

Comparisons were also made for chapters of ICD‐10 representing large categories of injuries/diseases in a similar manner. If two or more injuries/diseases are included in a single claim, the handling mode in the Statistics of Medical Care Activities in Public Health Insurance is unknown, and the following counting method is therefore used, as it is expected that this will limit the bias to the JMDC numbers. In this method, in the case of claims that have one or more principal injuries/diseases, the value counted is obtained by sharing 1 equally between all the principal injuries/diseases, whereas in the case of claims with no principal injuries/diseases, the value counted is obtained by sharing 1 equally between all the injuries/diseases. The comparison results for each chapter of ICD‐10 are shown in Figures 6, 7, 8.

**FIGURE 9:**
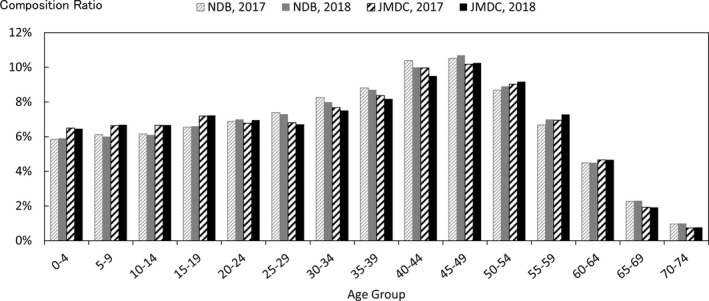
Comparison between age‐group composition ratios of data from health insurance societies and publicly available data (health‐insurance‐society insurance only)

**FIGURE 10:**
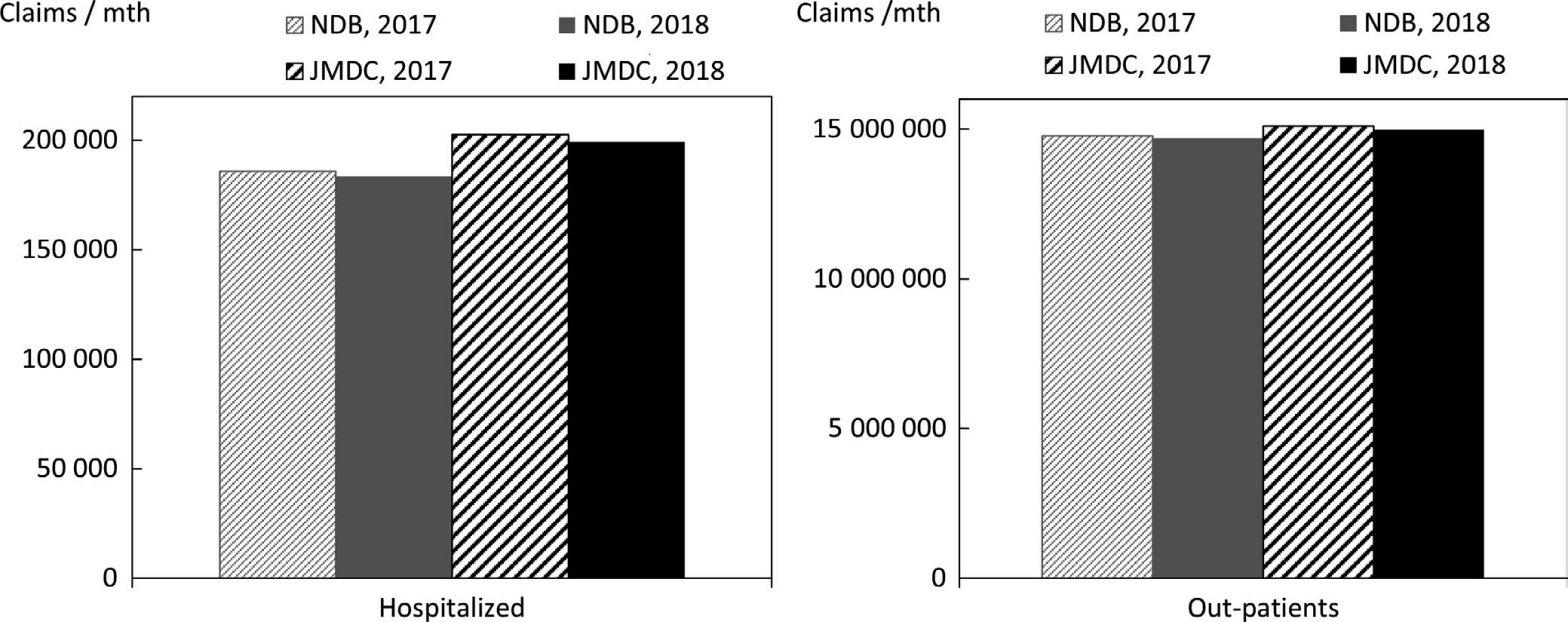
Comparison between scaled‐up data from health insurance societies and publicly available data (health‐insurance‐society insurance only)

**FIGURE 11:**
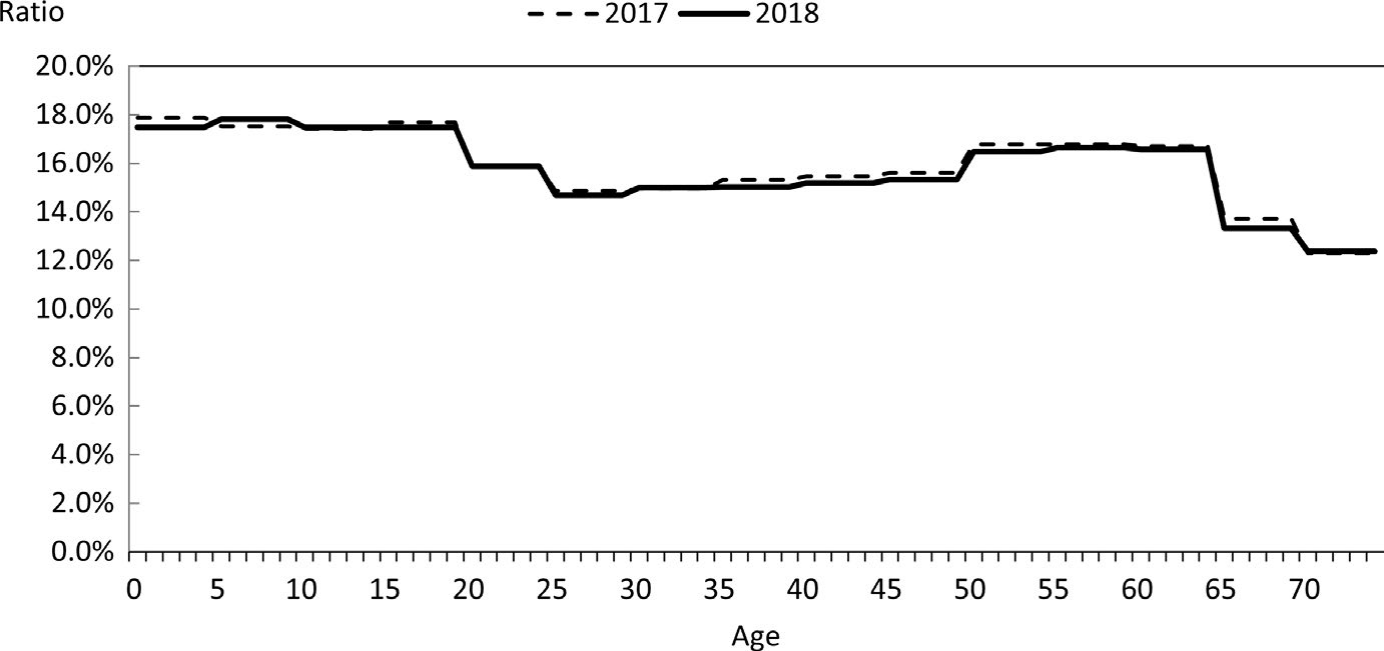
Ratios used for comparison of data from health insurance societies relative to publicly available data (health‐insurance‐society insurance only) (database population relative to total population of health‐insurance‐society insurance only)

In addition, taking the health insurance societies as the data source, JMDC data and publicly available data of health insurance societies were compared. Figure 9 shows the results of comparison between the age‐group composition ratios of this database and the numbers in the column of health‐insurance‐society insurance in Table 1 (Age‐group composition of total population and persons with health insurance) of the MHLW's Fact‐Finding Study of Persons with Health Insurance and Seamen's Insurance at the beginning of October 2017 and October 2018. Figure 10 shows the results of comparison between (a) the number of health‐insurance‐society insurance claims in the June 2017 and June 2018 assessment in the Statistics of Medical Care Activities in Public Health Insurance and (b) the number of medical treatment claims for May 2017 and May 2018 in this database, with both these numbers scaled up by the relative numbers of people of each gender and age‐group in the population insured by health insurance societies and the insured person population of this database as of the beginning of October 2017 and October 2018. The publicly available data for persons insured by health insurance societies were calculated by multiplying the total of insured persons and dependents (at October 1, 2017 and October 1, 2018) in Table 1 of Health‐insurance‐society Insurance Section (Insured by age and gender, number of insured persons, dependent gender, number of dependents, dependent rate, average standard monthly remuneration, average standard bonus and average total remuneration) in the MHLW's Fact‐Finding Study of Persons with Health Insurance and Seamen's Insurance, by health‐insurance‐society insurance (at October 1, 2017 and October 1, 2018), shown in Table 1 of Summary of Survey Results Section (Age‐group composition of total population and persons with health insurance) of the MHLW's Fact‐Finding Study of Persons with Health Insurance and Seamen's Insurance. The ratios used for scaling up are shown in Figure 11.

## 5 DATA RESOURCE ACCESS

JMDC makes its database of data from health insurance societies widely available, on a fee‐paying basis, for use of surveys, research, and commercial purposes like the databases sourced from medical institutions JMDC created.^1^ Inquiries about this database can be made via JMDC's website (https://www.jmdc.co.jp/en/bigdata), and it can be accessed after completing the contract for use of specific data.

## 6 PROFILE IN A NUTSHELL

1. JMDC has created a database from data on medical expenses, etc., collected from health insurance societies.
2. The earliest data that can be accessed are for January 2005. The number of persons whose data can be accessed for each month tends to increase from the initiation. Currently (the end of June 2020), the number of persons included in the database of data from health insurance societies, including those withdrawn at some point, is approximately 9.8 million.
3. In principle, all data collected from health insurance societies are included in the databases. Non‐standardized data are standardized using a dictionary, and the database permits data to be followed in a time‐series on the basis of anonymized personal IDs.
4. The database of data from health insurance societies includes information about insured persons, medical institutions, claims, and health checkup results.
5. JMDC makes this database widely available, on a fee‐paying basis, for use for surveys, research, and commercial purposes. Inquiries about this database can be made via JMDC's website (https://www.jmdc.co.jp/en/bigdata), and they can be accessed after completing the contract for use of specific data.

## 7 CONCLUSION

The database of data from health insurance societies is unique for Japan and has the following characteristics: (a) the basic population can be ascertained; (b) standardization is carried out using a dictionary; and (c) anonymized individual IDs can be followed on the basis of a time‐series over various periods, with the earliest starting date being January 2005. However, the database of data from health insurance societies, owing to the properties of the data source, has certain limitations, in that the disease status and test results cannot be ascertained, and there is insufficient access to data for elderly people. Use of this database is on a fee‐paying basis. The characteristics mean that it is provided for a wide range of purposes.

## CONFLICT OF INTEREST

This article was co‐authored by academic researchers and JMDC Inc. (JMDC). First four authors are affiliated with JMDC. The other authors have no connection with JMDC.

## ETHICAL APPROVAL AND INFORMED CONSENT

The study about objective descriptions of the database was approved by the Ethics Committee of the Research Institute of Healthcare Data Science (RIHDS) (Date of approval, September 11, 2020; Approval number, RI2020011). Informed consent is not applied for this study because personal information is not handled in it.
